# Structural Insights into the Inhibition of Actin-Capping Protein by Interactions with Phosphatidic Acid and Phosphatidylinositol (4,5)-Bisphosphate

**DOI:** 10.1371/journal.pcbi.1002765

**Published:** 2012-11-01

**Authors:** Roman Pleskot, Přemysl Pejchar, Viktor Žárský, Christopher J. Staiger, Martin Potocký

**Affiliations:** 1 Institute of Experimental Botany, Academy of Sciences of the Czech Republic, Prague, Czech Republic; 2 Department of Plant Physiology, Charles University in Prague, Prague, Czech Republic; 3 Department of Biological Sciences, Purdue University, West Lafayette, Indiana, United States of America; Peking University, China

## Abstract

The actin cytoskeleton is a dynamic structure that coordinates numerous fundamental processes in eukaryotic cells. Dozens of actin-binding proteins are known to be involved in the regulation of actin filament organization or turnover and many of these are stimulus-response regulators of phospholipid signaling. One of these proteins is the heterodimeric actin-capping protein (CP) which binds the barbed end of actin filaments with high affinity and inhibits both addition and loss of actin monomers at this end. The ability of CP to bind filaments is regulated by signaling phospholipids, which inhibit the activity of CP; however, the exact mechanism of this regulation and the residues on CP responsible for lipid interactions is not fully resolved. Here, we focus on the interaction of CP with two signaling phospholipids, phosphatidic acid (PA) and phosphatidylinositol (4,5)-bisphosphate (PIP_2_). Using different methods of computational biology such as homology modeling, molecular docking and coarse-grained molecular dynamics, we uncovered specific modes of high affinity interaction between membranes containing PA/phosphatidylcholine (PC) and plant CP, as well as between PIP_2_/PC and animal CP. In particular, we identified differences in the binding of membrane lipids by animal and plant CP, explaining previously published experimental results. Furthermore, we pinpoint the critical importance of the C-terminal part of plant CPα subunit for CP–membrane interactions. We prepared a GST-fusion protein for the C-terminal domain of plant α subunit and verified this hypothesis with lipid-binding assays *in vitro*.

## Author Summary

The actin cytoskeleton is a prominent feature of eukaryotes and plays a central role in many essential aspects of their lives. This highly malleable structure responds to a wide range of stimuli with rapid changes in organization or dynamics. These responses are thought to be mediated by dozens of actin-binding proteins, the biochemical activities of which have been demonstrated to be tightly controlled by other proteins and/or signal transduction mediators. In this study, we investigated the structural aspects of inhibition of actin-capping protein (CP) by phosphatidic acid (PA) and phosphatidylinositol (4,5)-bisphosphate (PIP_2_). We employed diverse computational methods in combination with experimental approaches to reveal mechanistic details of the direct interaction of CP with the phospholipid membrane containing either PA or PIP_2_. Importantly, we found several differences between PA/PIP_2_–CP interactions from two distinct species, Arabidopsis and chicken, that enable us to explain and expand upon previously published results. Our new data shed light on the nature of interactions between peripheral membrane proteins and PA-containing lipid bilayers. In addition to a description of the phospholipid-mediated regulation of CP activity, our work also significantly contributes to the ongoing debate on structural details of protein interactions with phospholipids.

## Introduction

The actin cytoskeleton represents part of a complex network that is essential for cell motility, organelle movements and cell polarity. Actin filaments are dynamic structures in general and, in plant cells, they serve as tracks for some of the fastest movements on earth. To regulate actin cytoskeleton organization and dynamics, cells use more than a hundred classes of actin-binding proteins (ABPs). To a limited extent, these proteins can be classified based on their binding properties and activities *in vitro*. Some ABPs bind actin monomers regulating the size and activity of the polymerizable actin pool, whereas others bind to the sides of actin filaments. Side-binding proteins can create higher-order filament structures like meshworks and bundles, or they can create breaks and sever filaments. Another group of ABPs interacts with actin filament ends and regulates the stability and dynamics of polymer assembly/disassembly [1]. A conserved member of this latter group is actin-capping protein (CP or CapZ), which inhibits the addition and loss of actin subunits at the barbed end of actin filaments [2], [3].

CP is a heterodimeric protein with a mushroom-like structure [4]. Each monomer, α and β subunit (CPα and CPβ), has a molecular weight of approx. 30 kDa and despite their sequence divergence, they have similar structural folds [4]. Several recent studies describe a mode of interaction between CP and the actin filament barbed end [5], [6], highlighting the importance of C-terminal domains from both subunits. These C-terminal parts form so-called tentacles laying on the top of the protein and are mainly composed from amphipathic helices [4]. It has been shown previously that binding of CP to actin filaments is regulated by several other proteins, either by competition for filament ends or by direct protein-protein interactions and allosteric regulation [7]. Another set of key regulators that inhibit CP activity are the signaling phospholipids, phosphatidylinositol (4,5)-bisphosphate (PIP_2_) and phosphatidic acid (PA) [8]–[12].

Phospholipids are part of the complex lipid-signaling language of eukaryotic cells and enable communication between plasma membrane, endomembrane compartments and cytoplasm. The role of phosphoinositides (PPIs) as signaling molecules was established many years ago [13]. More recently, PA has emerged as an important signaling messenger, especially in plant responses to biotic and abiotic stress [14]. This acidic phospholipid often functions by recruiting effector proteins to membranes in a spatio-temporally specific manner and/or it affects the biophysical properties of membranes [15]. One characteristic feature of PA and PPIs is their rapid turnover, which is mediated by particular enzymes producing and degrading them [16]. Despite the fact that both PA and PIP_2_ have important signaling functions, they significantly differ in their biophysical properties. PIP_2_ contains a bulky headgroup, with net charge ranging from −3 to −5 under physiological pH. and an inverted conical shape that promotes positive curvature of membranes. On the other hand, PA has a tiny headgroup with net charge ranging from −1 to −2 and it may induce formation of membrane structures with negative curvature [17], [18]. Although PIP_2_ binding by proteins is generally very well described and diverse binding-domains have been discovered [19], [20], much less is known about PA-protein interactions [14].

The ability of PIP_2_ to regulate CP has been known for a long time [7]; however, there is still some controversy about the exact binding site on CP. Kim *et al.* [11] performed an exhaustive site-directed and truncation mutagenesis of chicken CP (GgCP). These authors report that mutation of basic amino acids located on the α tentacle (R256, K260) as well as on the β subunit (R225) caused a reduction in PIP_2_ binding by about 4-fold. A similar reduction in PIP_2_ binding was observed following deletion of the last 28 C-terminal residues from the α tentacle. Although these results clearly show the importance of the α tentacle for binding to phospholipids, neither mutations or truncations totally abolished PIP_2_ binding. Kuhn and Pollard [12] studied fission yeast CP and its interactions with PPIs. These authors did not find any effect of various PPIs, including PIP_2_, on *Schizosaccharomyces pombe* CP activity. They constructed a homology model for CP from several species and, based on the comparison of electrostatic potentials mapped onto these structures, they hypothesize that a positively-charged patch located on CPβ close to the basic cluster on the α tentacle (which is absent in *S. pombe* CP) also contributes to the interaction with PPIs. Identification of a PA-binding site on CP remains more elusive; two seminal works that describe the effect of signaling phospholipids on mammalian CP, indicate that PA is not able to inhibit and/or dissociate this protein from actin filaments [8], [9]. However, we showed that mouse CP was able to bind PA, but with lower affinity than *Arabidopsis thaliana* CP (AtCP). We also demonstrated that PA is a potent inhibitor of AtCP activity, preventing it from interacting with filament barbed ends [10].

In this study, we focus on the interaction between AtCP, GgCP, PA and PIP_2_ in the context of phospholipid bilayers. To gain a structural perspective about these interactions, we utilized a combination of different computational methods and experimental approaches. We used the recently described MARTINI force field [21], [22] to investigate dynamics of CP binding to phospholipid bilayers containing PA or PIP_2_. We show different preferences of animal and plant CP towards distinct signaling phospholipids. Our results clearly reveal the importance of C-terminal tentacles from both subunits in these interactions. We further confirm the importance of the α subunit tentacle from AtCP in the PA interaction with an *in vitro* binding experiment using a GST-fusion protein. Altogether, our results explain and significantly expand upon previously published results [10]–[12].

## Results

### CP is widely distributed across eukaryotes

**Figure 1:**
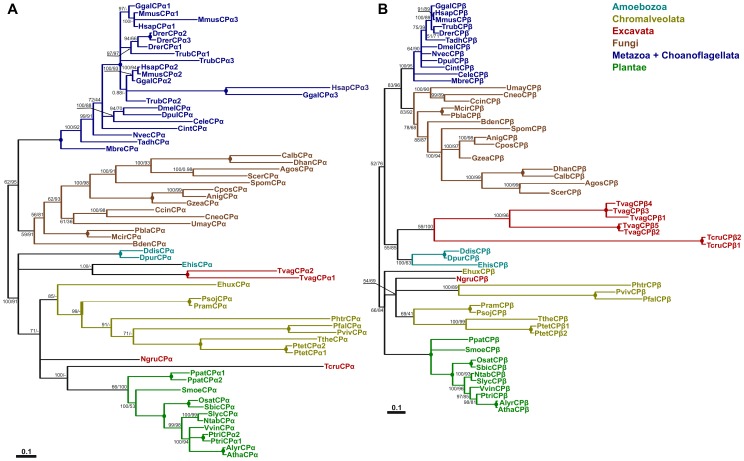
Phylogenetic analysis of CPα (A) and CPβ (B). Both trees represent protein bayesian phylogeny of particular genes. Numbers at nodes correspond to posterior probabilities from Bayesian analysis and the approximate likelihood ratio test with SH-like (Shimodaira-Hasegawa-like) support from maximum likelihood method, respectively. Circles represent support 100% by both methods, Missing values indicate support below 50%, dash indicates that a different topology was inferred by ML method. Branches were collapsed if inferred topology was not supported by both methods. Scale bar indicating the rates of substitutions/site is shown in corresponding tree. Abbreviations used: Agos – *Ashbya gossypii*, Alyr – *Arabidopsis lyrata*, Anig – *Aspergillus niger* Atha – *Arabidopsis thaliana*, Bden – *Batrachochytrium dendrobatidis*, Calb – *Candida albicans*, Ccin – *Coprinopsis cinerea*, Cele – *Caenorhabditis elegans*, Cint – *Ciona intestinalis*, Cneo – *Cryptococcus neoformans*, Cpos – *Coccidioides posadasii*, Ddis – *Dictyostelium discoideum*, Dhan – *Debaryomyces hansenii*, Dmel – *Drosophila melanogaster*, Dpur – *Dictyostelium purpureum*, Dpul – *Daphnia pulex*, Drer - *Danio rerio*, Ehis - *Entamoeba histolytica*, Ehux - *Emiliania huxleyi*, Ggal – *Gallus gallus*, Gzea – *Gibberella zeae*, Hsap – *Homo sapiens*, Mbre – *Monosiga brevicollis*, Mcir – *Mucor circinelloides*, Mmus – *Mus musculus*, Ngru – *Naegleria gruberi*, Ntab – *Nicotiana tabacum*, Nvec – *Nematostella vectensis*, Osat – *Oryza sativa*, Pbla – *Phycomyces blakesleeanus*, Pfal – *Plasmodium falciparum*, Phtr – *Phaeodactylum tricornutum*, Ppat – *Physcomitrella patens*, Pram – *Phytophthora ramorum*, Psoj – *Phytophthora sojae*, Ptet - *Paramecium tetraurelia*, Ptri – *Populus trichocarpa*, Pviv – *Plasmodium vivax*, Sbic – *Sorghum bicolor*, Scer – *Saccharomyces cerevisiae*, Slyc – *Solanum lycopersicum*, Spom – *Schizosaccharomyces pombe*, Smoe – *Selaginella moellendorffi*, Tadh – *Trichoplax adherans*, Tcru – *Trypanosoma cruzi*, Trub – *Takifugu rubripes*, Tthe – *Tetrahymena thermophila*, Tvag – *Trichomonas vaginalis*, Umay – *Ustilago_maydis* and Vvin – *Vitis vinifera*.

Given that CP has been identified as one of the major regulators of actin dynamics in different species, such as animals, fungi and plants [7], we asked whether CP is a generally distributed actin-regulating protein in eukaryotes. To achieve this goal, we searched more than 50 genomes for different species covering members of almost all eukaryotic superkingdoms [23]. Both CP subunits are well conserved in most eukaryotic lineages and are mostly present as single-copy genes. Nevertheless, in some organisms CP genes are multiplied; for example, vertebrates have three different genes for the α subunit and *Trichomonas vaginalis* has five genes for the β subunit (Figure 1). Moreover, the vertebrate gene for β subunit undergoes alternative splicing, producing additional variability [7]. It is worth noting that there is no organism with just one subunit gene for the heterodimer, i.e. an α gene but no β gene, or *vice versa*; this finding correlates well with genetic and biochemical data indicating strict dependency between α and β subunits. Surprisingly, we have not found CP genes for either subunit in sequenced genomes of green algae, red algae and in certain parasites such as *Toxoplasma gondii*. Some of these organisms probably lost CP genes during evolution, mainly because of their life strategies, i.e. parasites or extremophiles. The overall phylogeny of both CP subunits mainly follows organismal evolution (Figure 1). Metazoan genes, together with Choanoflagellate *Monosiga brevicollis* as a basal clade, cluster with Fungi in the case of both CP subunits. Plant sequences also form well supported groups. The phylogenetic relationships between other sequences of CPα (from Chromalveolata, Excavata and Amoebozoa groups) are not so clear. In the case of CPβ, Ameobozoa and Excavata sequences form well supported clusters. We also tried to find homologs of the eukaryotic protein in eubacteria and archeabacteria using more sensitive search tools, such as PSI-BLAST [24], but we did not found any obvious homologous sequences. Therefore, it is reasonable to speculate that CP is an eukaryotic innovation, similar to other ABPs, e.g. formins [25].

### Prediction of a PA/PIP_2_-binding site from a homology model of *Arabidopsis* CP

**Figure 2:**
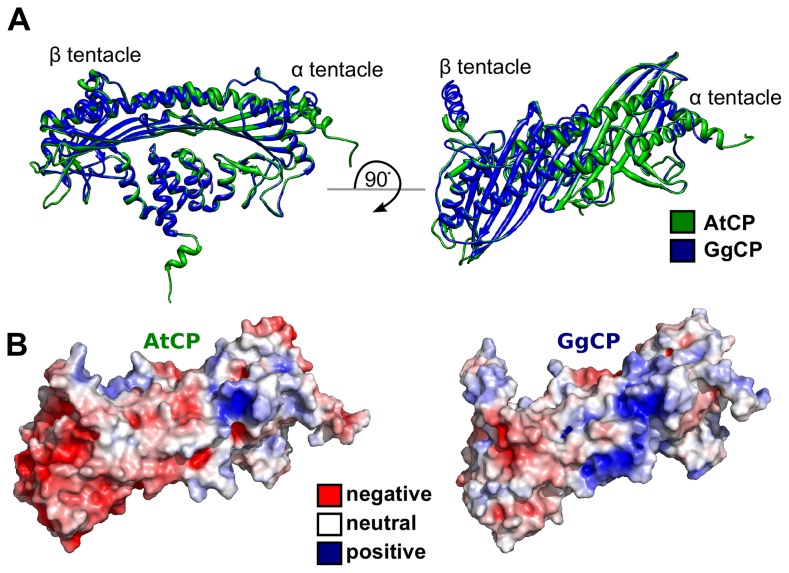
Structural comparison of AtCP and GgCP. **A** Superimposition of the homology-model for plant AtCP (in green) on the X-ray structure of chicken GgCP (in blue). **B** Electrostatic potential mapped on the structure of AtCP and GgCP ranging from −5 (red) to +5 (blue) kbT/e_c_. This figure was prepared with the UCSF Chimera package [53].

To clarify the mode of animal CP binding to PIP_2_ and to compare it with the binding of CP from different species to PA and PIP_2_, we utilized diverse methods of computational structural biology. First, we constructed a homology model for AtCP using the crystal structure of GgCP α1β1 (also known as CapZ; [4]) as a template (Figure 2A). A comparison of electrostatic surface potential for both structures shows marked differences in the distribution of charged residues. AtCP is much more negatively charged than the chicken protein (Figure 2B), but it contains one positively charged patch corresponding to the PIP_2_-binding region on GgCP identified by Kim *et al.* [11]. To further test the binding modes between PA and PIP_2_ binding by AtCP and GgCP, we used a computational molecular docking approach similar to that of Kim *et al.* [11]. Results for the docking of truncated PA (diacetyl-PA) to AtCP ended with a single prediction of binding site and correlate well with the positively-charged patch located on the α tentacle (Figure S1). We also computed the docking of a truncated PIP_2_ molecule to AtCP with the same results. As a control for these experiments, we used phosphatidylcholine (PC) and docking of this molecule did not result in any single prediction.

### Molecular dynamics simulations reveal binding modes for CP and PA/PIP_2_ lipid bilayers

**Figure 3:**
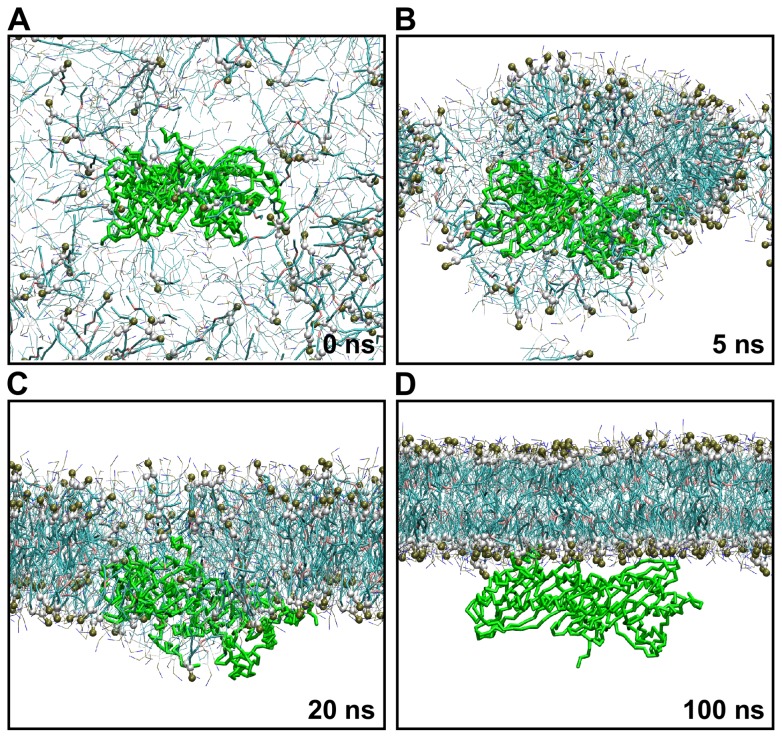
Self-assembly of lipid bilayer in the presence of AtCP. Self-assembly CG-MD simulation of membrane containing 20% POPA (charge −2)/POPC at time **A** 0 ns, **B** 5 ns, **C** 20 ns, and **D** 100 ns. CG water molecules and Na^+^ ions are not shown for the sake of clarity. Headgroups and glycerol backbone atoms of POPA are highlighted in van der Waals representation. Only protein backbone atoms are shown in licorice representation. This figure was prepared using VMD [54].

Phospholipids spontaneously form more complex systems, such as membranes or vesicles; therefore, we thought it important to ask what is the mode of CP binding to signaling phospholipids in the context of a lipid bilayer. Molecular dynamics (MD) simulation provides a useful and powerful tool to study complex biological systems, such as membranes or proteins [26]–[28]. We employed coarse-grained MD (CG-MD) with the MARTINI force field [21], [22]; this allowed us to simulate larger systems for longer periods of time and has been successfully applied to describe processes like raft-like structure formation, membrane protein dynamics or SNARE-mediated vesicle fusion [28]–[30]. We modeled self-assembly of a lipid bilayer in the presence of CP protein, as this procedure has been shown to be advantageous for the characterization of peripheral membrane protein dynamics [31], [32]. Specifically, we simulated several systems comprising different concentrations of 1-palmitoyl-2-oleoyl-phosphatidic acid/1-palmitoyl-2-oleoyl-phosphatidylinositol (4,5)-bisphosphate and 1-palmitoyl-2-oleoyl-phosphatidylcholine (POPA/POPIP_2_ and POPC) in the presence of AtCP or GgCP (Table 1). Snapshots from 100 ns of self-assembly of a lipid bilayer containing 20% POPA in POPC in the presence of AtCP are shown in Figure 3. We observed formation of a lipid bilayer within approx. 30 ns in all simulations. This is similar to the time required for membrane formation as described by previous studies [32], [33]. The membrane initially aggregates in the vicinity of CP (Figure 3B); however, the protein is very quickly pushed from the core of the lipid bilayer (Figure 3C, D). CP is peripherally bound to the membrane after approx. 50 ns and remains closely attached to the membrane for an additional 50 ns (Figure 3D). In all simulations performed (i.e. either AtCP or GgCP, and either POPA or POPIP_2_ in POPC membranes), the CP protein faces towards the lipid bilayer via its tentacles (Figure 3D), but the involvement of the tentacles in the interaction with the membrane is slightly different for particular simulations. Importantly, the protein always ends in this position independent of its initial orientation in the simulation box.

**Table 1:** Summary of the simulated systems with wild-type protein.

System	The region of CP involved in the interaction with phospholipid bilayer*
**AtCP**	
50% POPA (−1)/POPC	α tentacle
20% POPA (−1)/POPC	no binding
20% POPA (−2)/POPC	α tentacle
10% POPA (−2)/POPC	no binding
5% POPIP_2_/POPC	α tentacle, β tentacle
1% POPIP_2_/POPC	loose binding
**GgCP**	
20% POPA (−2)/POPC	β tentacle
5% POPIP_2_/POPC	α tentacle, β tentacle
1% POPIP_2_/POPC	α tentacle
POPC	no binding

* All simulations were run for 500 ns and repeated 3–5 times with different initial velocities.

**Figure 4:**
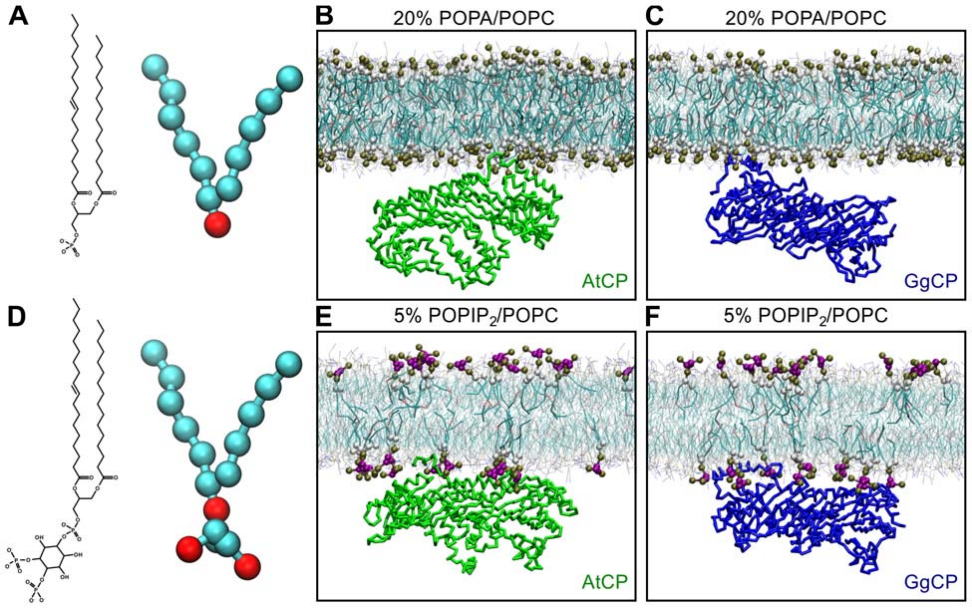
Comparison of interaction of AtCP and GgCP with distinct membranes at 500 ns. Chemical diagrams and CG representations of **A** POPA and **D** POPIP_2_. The final state of the MD system containing **B** AtCP – 20% POPA (charge −2)/POPC, **C** GgCP – 20% POPA (charge −2)/POPC, **E** AtCP – 5% POPIP_2_/POPC and **F** GgCP – 5% POPIP_2_/POPC. CG water molecules and Na^+^ ions are not shown for the sake of clarity. Headgroups and glycerol backbone atoms of POPIP_2_ and POPA are highlighted in van der Waals representation. AtCP is colored green and GgCP is blue; only backbone atoms are shown in licorice representation. This figure was prepared with VMD [54].

**Figure 5:**
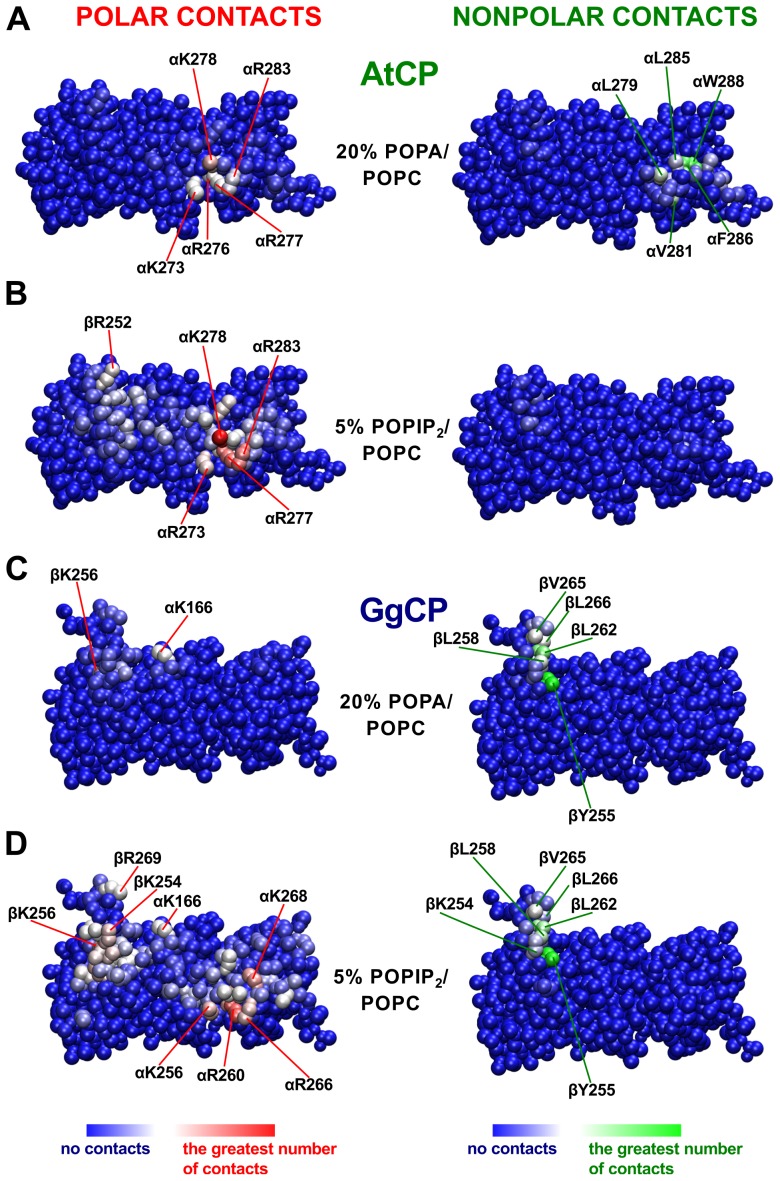
Polar and nonpolar contacts of AtCP (A,B) and GgCP (C,D) with distinct membranes. Polar contacts were defined as the number of POPA/POPIP_2_ headgroup atoms within 8 Å of protein atoms. Nonpolar contacts were defined as the number of POPA/POPIP_2_ and POPC tail atoms within 8 Å of protein atoms. Contacts represent the average number computed for each performed simulation over last 200 ns. This figure was prepared using VMD [54].

After 500 ns of simulation, clear differences in the binding mode between AtCP and GgCP proteins and the POPA/POPC lipid bilayer were observed (Figure 4 and Figure S2). We found that the binding of AtCP to membranes composed from POPA/POPC is dependent on the concentration of POPA and on the PA charge, −1 or −2. In the case of POPA with a charge −1, AtCP only binds membranes with a high content of POPA (50%). By contrast, AtCP binds to membranes comprising 20% POPA with the charge −2 (Figure 4B), but not to 10% POPA. In all positive cases, AtCP binds the membrane via the α tentacle (Figure 4B and Figure S2A). Moreover, and in good agreement with docking results, residues from the positively-charged patch of the α tentacle (K273, R276, K277, K278 and R283) interact with POPA (Figure 5A). Furthermore, the amphipathic helix at the very end of the α tentacle is embedded in the membrane (Figure 4B) via its hydrophobic residues (Figure 4B, L279, V281, L285, F286 and W288). On the other hand, GgCP binds membranes containing POPA solely via the β tentacle (Figure 4C) and interacts with the membrane mainly by nonpolar contacts (Figure 5C).

To study the mode of CP binding to POPIP_2_/POPC membranes, we used two different concentrations of POPIP_2_ (1 and 5%). AtCP interacts with 5% POPIP_2_ membranes with both tentacles (Figure 4E) and, similarly to POPA, the majority of polar interactions are mediated by the positively-charged region on the α tentacle (Figure 5B). However, we observed a decreased number of nonpolar contacts between AtCP and membranes containing 5% POPIP_2_/POPC (Figure 5B) compared to 20% POPA/POPC (Figure 5A). This correlates very well with density profiles computed for these two simulated systems (Figure S3), where we found that the α tentacle is much more embedded into the hydrophobic part of the phospholipid bilayer comprising 20% POPA/POPC. Intriguingly, we did not found any preferential binding site when simulating AtCP with membranes containing 1% POPIP_2_ but rather observed that protein rotates closely to the membrane (Figure S2B). Conversely, we observed GgCP binding to membranes with both concentrations of POPIP_2_ (Table 1). The interaction of GgCP with membranes containing 5% POPIP_2_/POPC is mediated by both tentacles (Figure 4F and Figure 5D). Interestingly, we observed that the binding is mediated just by the α tentacle when we used a lower amount of POPIP_2_ in the membrane (1%, Figure S2C). We also performed self-assembly simulations and subsequent extension for conditions without any signaling lipid in the membrane; in this case we did not observe any binding between CP and POPC bilayers (Figure S2D).

In summary, we observed that AtCP differs from its vertebrate counterpart GgCP in the way it interacts with membranes containing POPA/POPC or POPIP_2_/POPC (Table 1). The interaction between AtCP – POPA/POPC membrane is mediated solely by the α tentacle and the binding is provided by the combination of polar and nonpolar interactions (Figure 4B and Figure 5A). On the other hand, GgCP interacts with the lipid bilayer containing POPA/POPC with the β tentacle and the interaction seems to be mediated preferentially by nonpolar contacts (Figure 4C and Figure 5C). The interaction of either AtCP or GgCP with the membrane consisted of POPIP_2_/POPC is mediated by both tentacles (Figure 4E and 4F), although there are also significant differences in the POPIP_2_ binding by AtCP and GgCP. In particular, the longer β tentacle of GgCP provides more nonpolar contacts with the POPIP_2_-containing bilayer in comparison with AtCP (Figure 5B and 5D).

### *In silico* mutation of PA-binding residues disrupts the membrane-association of AtCP

**Figure 6:**
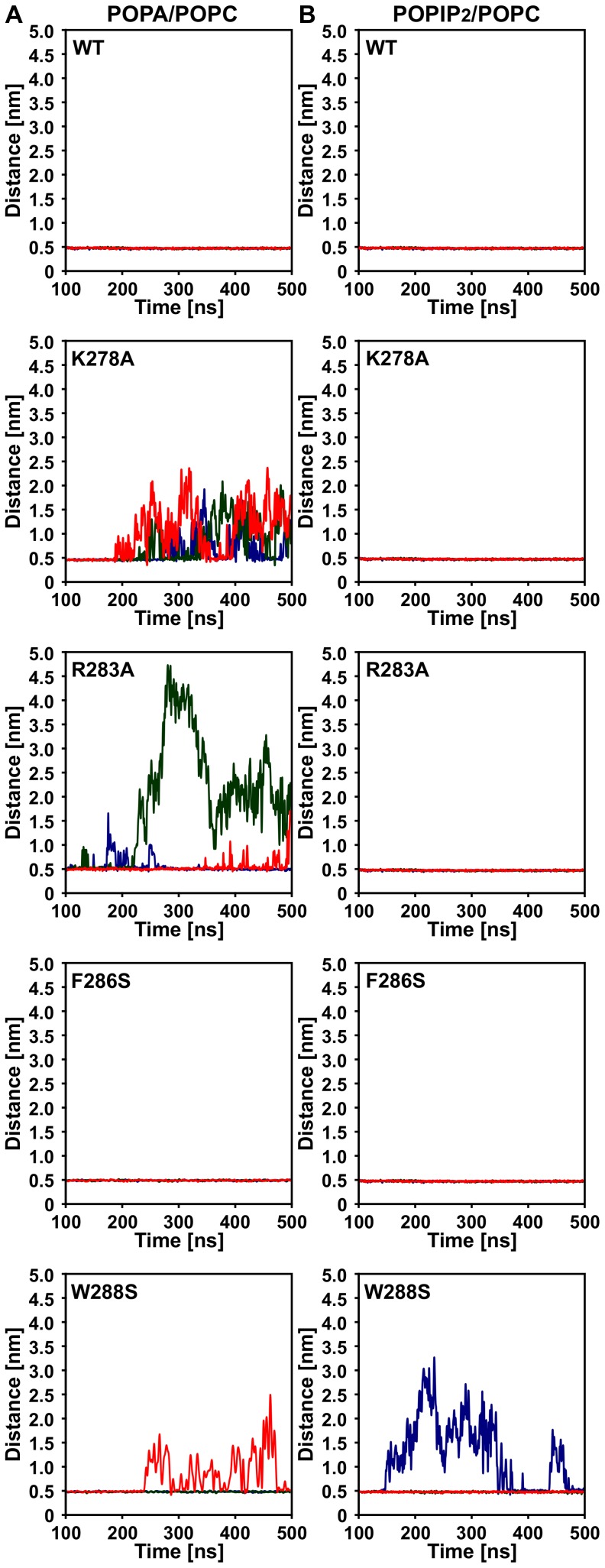
Effects of mutations in AtCP on its membrane association. Time-course for three independent simulations with wild-type (WT) AtCP and several different mutations is shown as the distance of the center of mass of the protein from the center of mass of the bilayer. **A** System with 20% POPA/POPC. **B** System with 5% POPIP_2_/POPC.

To further confirm the importance of the α tentacle for association of AtCP with POPA/POPC membranes, we performed *in silico* mutagenesis of two residues with the greatest number of polar (CPα-K278A and CPα-R283A) as well as for the two most important nonpolar contacts (CPα-F286S and CPα-W288S). We simulated three 500 ns runs of CG-MD as described above and computed minimal distances between AtCP and membrane during these simulations. As shown in Figure 6A, wild-type AtCP always remains closely associated with the membrane. On the other hand, mutation of the polar residue K278 to alanine leads to complete disruption of AtCP-POPA/POPC association. Similar but weaker effects can be observed for the CPα-R283A mutation. Interestingly, CPα-W288S mutation was also able to disrupt binding of AtCP to the POPA/POPC membrane, although not in every run. On the other hand, we did not observe any effect caused by mutation of CPα-F286S. We also performed analogous simulations for the mutated AtCP proteins with POPIP_2_/POPC membranes (Figure 6B). In this case, we found that only mutation of W288 has an effect on the association of AtCP with the membrane. Collectively, these results further confirm the critical importance of the CP α tentacle for PA binding that is mediated by interaction site containing positively charged residues K278 and R283. The effect of the W288S mutation on both POPA/POPC and POPIP_2_/POPC-binding supports the hypothesis of structural importance of W288 (homologous to W271 in GgCP) for stability of the α tentacle as proposed by Kim et al [6].

### Quantitative aspects of the CP interaction with PA/PIP_2_

**Figure 7:**
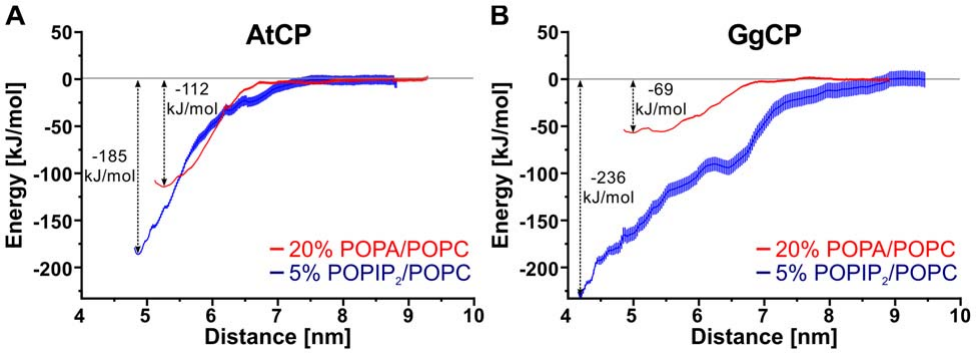
Potential of mean force (PMF) curves for pulling AtCP (A) and GgCP (B) from distinct membranes. Red lines represent PMF curves for pulling respective protein from membranes containing 20% POPA/POPC. Blue lines represent PMF curves for pulling respective protein from membranes containing 5% POPIP_2_/POPC. Vertical red and blue lines indicate error bars generated by the Bayesian boostrap method of g_wham program [55].

Previously, we described dissociation constant (*K*_d_) values for plant and mouse CP binding to PA and PIP_2_ micelles, as analyzed by changes in endogenous tryptophan fluorescence [10]. The findings show that AtCP has a somewhat higher apparent affinity for PIP_2_ micelles than for PA (11 µM versus 17 µM, respectively). The apparent affinities of the animal protein for PA and PIP_2_ are markedly different, with mouse CP showing a higher affinity for PIP_2_ (8 µM for PIP_2_ versus 59 µM for PA). Here, we employed the potential of mean force (PMF) calculation with the umbrella sampling protocol [34] to gain insight into the quantitative aspects of the computed interactions. We used steered molecular dynamics to pull the protein away from the membrane and to generate sampling windows for PMF calculation. For this type of pulling experiment, we applied position restraints on the lipids to keep them in the membrane. Figure 7 shows PMF curves for four selected systems. We found that GgCP interacts most tightly with membranes containing 5% PIP_2_/POPC with ΔG −236 kJ/mol. AtCP interacts with membranes of the same composition with ΔG −185 kJ/mol. In comparison to GgCP (ΔG −69 kJ/mol), AtCP interacts more strongly with membranes composed from 20% POPA/POPC (ΔG −112 kJ/mol). Importantly, this is a similar trend compared to the experimental data; there is a huge difference between the binding of PA and PIP_2_ for GgCP and a much smaller difference in the case of AtCP.

### Sequence comparison of the α tentacles shows important differences between plant and vertebrate CP and their lipid-binding abilities

**Figure 8:**
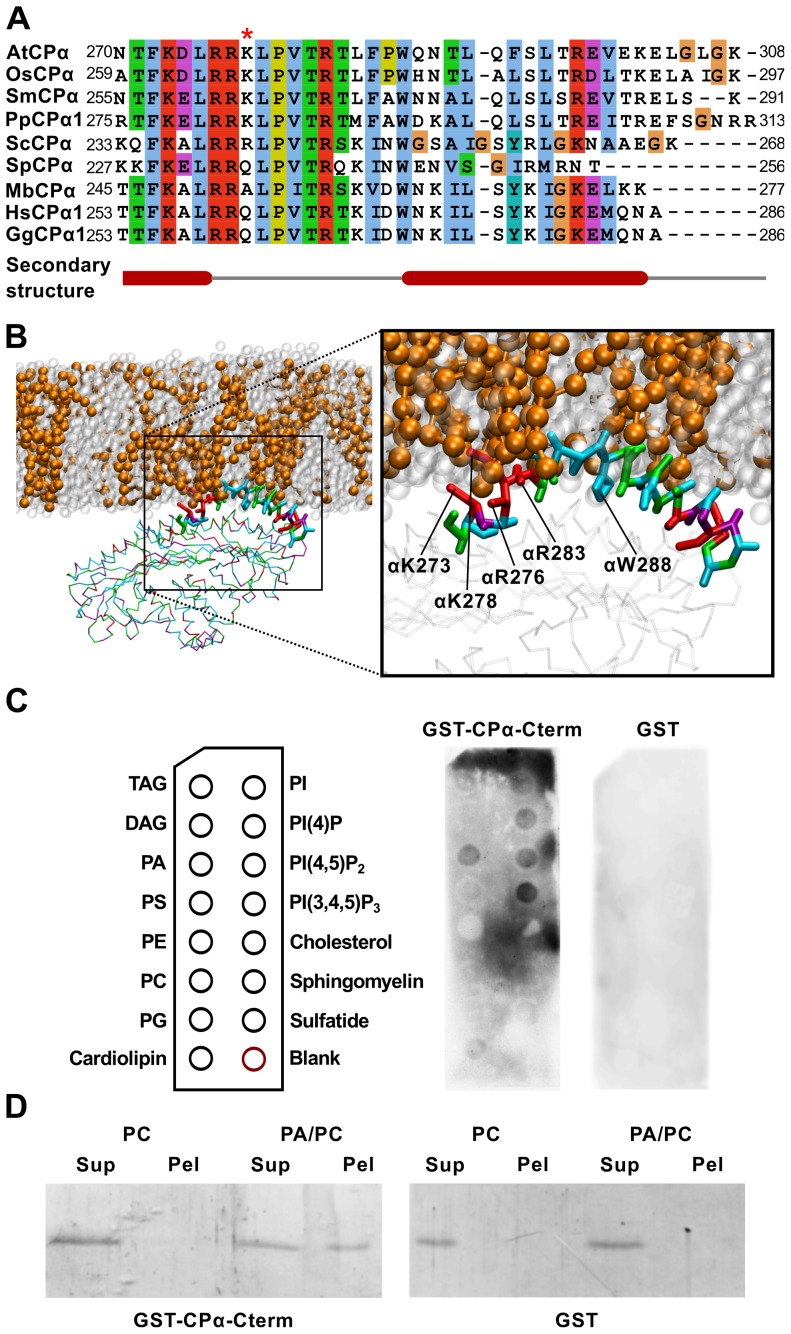
Details in the interaction of AtCP with the membrane containing phosphatidic acid. **A** Sequence comparison of C-terminal parts of CPα (CPα-Cterm) from different species. The mafft algorith [40] was used to construct multiple alignments and the final figure was produced using the Jalview alignment editor [56]. Abbreviations used: At – *Arabidopsis thaliana*, Gg – *Gallus gallus*, Hs – *Homo sapiens*, Mb - *Monosiga brevicollis*, Os – *Oryza sativa*, Pp – *Physcomitrella patens*, Sc - *Saccharomyces cerevisiae*, Sm - *Selaginella moellendorffii*, Sp - *Schizosaccharomyces pombe*. Red asterisk marks conserved Lys in plants. **B** A detailed view of AtCP interaction with membrane containing 20% POPA (charge −2)/POPC. This figure was prepared using VMD [54]. **C** Protein-lipid overlay assay for detecting interacting lipids. CPα-Cterm shows a preference for PA and PPIs. GST-CPα-Cterm bound to the lipids was detected by immunoblotting with an antibody against GST. Figure shows a representative result from 3 different experiments. **D** Liposome-binding assay of CPα-Cterm. PA binding was determined using 200 nm-sized vesicles containing 20% PA/PC or PC alone. After incubation of GST-AtCPα-Cterm with the vesicles, they were recovered by ultracentrifugation and protein bound was analysed by SDS-PAGE. As negative control, GST alone was used. Figure shows representative result from 4 different experiments.

A direct alignment of the primary sequences for the C-terminal tentacles from CP proteins across diverse eukaryotes (Figure 8A and Figure S4) revealed that although the positively-charged region located on the α tentacle is generally well conserved, several lineage-specific differences could be identified, which might explain distinct binding properties of AtCP and GgCP. Plant sequences generally have longer α tentacles (Figure 8A) with a conserved lysine (K278, in GgCP this is Q261), that shows the greatest number of polar contacts with PA (Figure 5A). Moreover, plant α tentacles contain leucine, proline and asparagine (L285, P287 and N290) instead of lysine, aspartate and lysine in vertebrate sequences (K268, D270 and K273), resulting in a decrease of polar residues in this region compared to animal CP. These amino acid changes facilitate the observed embedding of the plant α tentacle into PA-containing membranes (Figure 8B and Figure S3). Intriguingly, higher plants also have a shorter β tentacle and thus lack a major part of the amphiphatic helix located at this position in vertebrate CP (Figure S4).

### The PA-binding domain of plant CP is sufficient for lipid binding

To further confirm whether the AtCP α tentacle constitutes a PA-binding domain, we prepared a recombinant fusion protein between GST and the C-terminal 38 amino acids from AtCP α subunit (GST-CPα-Cterm). Protein-lipid overlay assays showed strong binding of the GST-CPα-Cterm to PA (Figure 8C), similar to our previous observations with full-length AtCP protein [10]. In addition, the interaction of GST-CPα-Cterm with a subset of PPIs including PIP_2_ and phosphatidylinositol (3,4,5)-trisphosphate (PIP_3_), as well as with cardiolipin and sulfatidate was also observed in this assay. Interestingly, cardiolipin and sulfatidate contain a phosphate/sulphate group and thus resemble PA and PPIs to some extent. However, the binding of PIP_3_, cardiolipin and sulfatidate to GST-CPα-Cterm is most probably non-physiological, as PIP_3_ is not present in plant membranes and cardiolipin is found only in bacteria and in the inner membrane of mitochondria. We also found that GST-CPα-Cterm binds to lipid vesicles containing 20% PA and PC in co-sedimentation experiments (Figure 8D). These two complementary approaches clearly demonstrate that the AtCP α tentacle is sufficient for PA binding.

## Discussion

We previously described different binding affinities for plant and animal CP interacting with two distinct signaling phospholipids, PA and PIP_2_ [10]. Here, we focused on the structural aspects of these interactions by employing diverse methods of structural bioinformatics. It has been shown that these methods, and particularly CG-MD simulation, can play a crucial role in our understanding of general principles of processes such as lipid bilayer formation, peptide segregation into raft-like structures in the membrane, and characterization of protein-lipid interactions with both integral- and peripheral-membrane proteins [28]. Recently, the combination of homology modeling and CG-MD was used to investigate interactions between diverse voltage sensors and lipid bilayers [35]. Initial all-atom MD studies done on GgCP, in the absence of membranes, revealed that the α tentacle is rather immobile and remains stationary on the protein surface during the simulation [36]. This immobility is mainly stabilized by the interaction of W271 of the amphiphatic helix with the core of the animal protein. Interestingly, we observed that the homologous tryptophan in AtCP (W288), together with other hydrophobic residues of the α tentacle, is embedded into the membrane after 500 ns MD simulation (Figure 8B). These data support the hypothesis of Wear and Cooper [37], that proposes the induction of α tentacle mobility by non-ionic detergent. We suggest that a lipid bilayer could have a similar effect on the mobility of the α tentacle and facilitate embedding of hydrophobic residues.

In this report, we describe differences between AtCP and GgCP for both C-terminal tentacles (Figure 8A and Figure S4), which may reflect distinct properties of CP–actin interaction between organisms. Alternatively, given that plant cells contain 10- to 100-fold lower amounts of PIP_2_ than PA [38], [39], one can speculate that differences in the tentacles is an adaptation to the distinct levels of PA and PIP_2_ in mammals and plants, i.e. increased binding properties of AtCP towards PA. As discussed above, we observed the embedding of the AtCP α tentacle into membranes containing PA. Consistent with this observation, we found a decreased number of polar residues in this tentacle. It is important to note that this difference is rather subtle, but mutations leading to a more nonpolar α tentacle could reduce actin binding [6]. We also observed that plant CPs have a shorter β tentacle and thus they lack the majority of the amphiphatic helix located in this region (Figure S4). We hypothesize that the PA- and actin-binding properties of plant CP have co-evolved to keep the right balance between actin regulation and responses to lipid signaling.

Kooijman et al. [18] described remarkable properties of PA and proposed a model for the electrostatics/hydrogen bond switch, where arginine and lysine residues on binding peptides can increase the charge of PA to −2. The authors also performed all-atom MD simulation of K_8_ and R_8_ peptides with bilayers formed from DOPC/DOPA and found that simulations where DOPA had charge −2, were in better agreement with experimental results. In our simulations, we observed the dependence of AtCP binding on the charge of PA, but it is important to note, that when we observed the interaction, the binding mode was very similar for each system regardless of the PA charge (Figure 4B and Figure S2A). Moreover, PA has a unique cone shape under physiological conditions and it has been proposed that PA could facilitate the insertion of hydrophobic protein domains into a bilayer [18]. Consistent with this hypothesis, we observed insertion of hydrophobic parts of the AtCP α tentacle into membranes containing PA (Figure 8B and Figure S3).

In our CG-MD simulations with membranes containing 5% POPIP_2_ and POPC, we observed the involvement of both tentacles with either animal or plant CP (Figure 4E, F and Figure 5B, D), suggesting cooperativity between both tentacles. When we simulated the system containing 1% POPIP_2_/POPC, we found that GgCP binds the phospholipid bilayer preferentially by the α tentacle (Table 1). Altogether, these results clearly show the importance of a positively-charged patch located on the α tentacle in both AtCP and GgCP. This region corresponds to lipid-binding site identified by Kim *et al.* [11]. We did not observe the involvement of the second putative PIP_2_-binding site proposed by Kuhn and Pollard [12]. Moreover, the latter positively-charged region is completely lacking in AtCP.

Importantly, we obtained very similar quantitative trends for the interactions studied herein when compared to experimental approaches [10]. We found a much smaller difference between the binding of PA and PIP_2_ by AtCP when compared to GgCP. The energies of the interactions computed from experimentally determined K_d_ values vary from −24 to −29 kJ/mol, whereas from the umbrella sampling protocol, we computed the energy ranging from −62 to −236 kJ/mol. These discrepancies could be explained by different composition of the membrane (experimental K_d_s were determined for the system with just one phospholipid, i.e. PA or PIP_2_, and the lipids were in micelles rather than bilayers).

The most recent information on CP–actin interactions comes from a study by Kim *et al.* [6], who combined computational approaches with a large scale site-directed mutagenesis. They propose a model in which GgCP interacts with actin mainly via its tentacles and faces the actin filament barbed end with the top of the mushroom structure. The authors identified 49 residues of mammalian CP (18 on CPα and 31 on CPβ). They mutated 45 of these residues and found that only 10 showed more than a 3-fold increase in K_d_. A direct comparison of these residues between GgCP and AtCP shows that 7 residues are highly conserved (these residues include CPα-E200, CPα-K256, CPα-R260, CPα-K268, CPβ-R195, CPβ-K223 and CPβ-R225 of mammalian CP). Interestingly, AtCP completely lacks nonpolar residues located on the β tentacle (L258, L262, L266) which are responsible for the interaction with the hydrophobic cleft in actin. In our computed modes of the CP-membrane interaction, we observed that CP binds membranes mainly via its tentacles. Therefore, it is tempting to speculate that steric hindrance imposed by CP–membrane binding prevents actin binding. Interestingly, GgCP bound to the PA-containing membrane has the α tentacle and the top of the mushroom-like structure unoccupied (Figure 4C). This could be an explanation why PA has not been described as an inhibitor of the activity of the animal CP [8], [9].

In summary, our results provide structural insight into the regulation of CP by two signaling phospholipids, PA and PIP_2_. A prominent role for the α and β C-terminal tentacles located on the top of the CP structure is apparent. We have shown differences of PA and PIP_2_ binding between AtCP and GgCP explaining published experimental data. Our results represent a comprehensive view of the interaction between CP and PA- or PIP_2_-containing membranes and reveal the mode of binding with structural implications for CP regulation. We also identified the PA-binding domain of AtCP and experimentally showed that it is sufficient for binding membranes *in vitro*. Our results call for intensive future research involving, in particular, a detailed mechanistic description of the phospholipid-induced uncapping of actin filaments. We also suggest that it would be relevant to examine the possible synergistic effects of distinct phospholipids on the inhibition of CP activity.

## Methods

### Sequence mining and analysis, multiple alignment, construction of phylogenetic trees

CP protein sequences were identified by gapped BLAST or PSI-BLAST [24] searching against the non-redundant protein database at the National Center for Biotechnology Information (http://blast.ncbi.nlm.nih.gov/Blast.cgi) using Arabidopsis annotated sequences with default settings. In addition, blast searches were conducted using Phytozome web page and DOE Joint Genome Institute (http://www.phytozome.net/; http://www.jgi.doe.gov/). In most cases, the search parameters were set at the default values; however, occasionally, modifications were used (the changed parameters included mostly length of the word and type of scoring matrice). Putative genes were initially identified based on the automatic annotation at the aforementioned databases. Since gene models based on computer annotations often contain errors, exon-intron structures were manually curated with the aid of experimentally-verified sequences or sequences from closely related species.

Multiple alignments were constructed with mafft algorithm (in einsi mode) [40] and manually adjusted. Maximum likelihood method using PhyML program [41] was employed for phylogeny inference with the WAG matrix, Γ-corrected among-site rate variation with four rate site categories plus a category for invariable sites, all parameters estimated from the data. Bayesian tree searches were performed using MrBayes 3.1 [42] with a WAG amino acid model, where all analyzes were performed with four chains and 1 000 000 generations per analysis and trees sampled every 100 generations. All four runs asymptotically approached the same stationarity after first 500 000 generations which were omitted from the final analysis. The remaining trees were used to infer the posterior probabilities for individual clades.

### Homology model

A homology model for AtCP was built on the X-ray structure for GgCP (rcsb 1IZN). The manually edited alignment obtained by PSIPRED [43] was used as input for MODELLER 9v8 [44]. As template contains shorter C-terminus of α subunit, residues ranging from 288 to 302 were forced to α-helix formation according to secondary structure prediction. The best model was selected on the energy and constraint violation values of MODELLER and further evaluated by PROSA and WHAT IF algorithms [45], [46]. APBS program [47] was used to compute electrostatic potential of CP.

### Molecular dynamics simulations

To simulate self-assembly of lipid bilayers in the presence of protein, the MARTINI CG force field was used [21], [22]. The protein was described according to ELNEDIN representation [48] with Rc 0.9 nm and K 500 kJ·mol^−1^·nm^−2^. CG model for POPIP_2_ molecule was prepared according to [49]. GROMACS 4.0.5 was used for all MD simulations [50]. Lenard-Jones and electrostatic interactions were shifted to 0 between 9 and 12 Å and between 0 and 12 Å, respectively. A relative dielectric constant of 15 was used. Simulations were run in NPT ensemble. The temperature of protein, lipids, and solvent was coupled separately at 310 K using the Berendsen algorithm, with a coupling constant 1.0 ps. The system pressure was coupled using the same algorithm with a coupling constant 3.0 ps, compressibility of 3·10^−5^ and reference pressure 1 bar. Simulations were performed using a 20 fs integration time step. The protein, lipids and water were placed randomly in the simulation box. Na^+^ ions were added to ensure electroneutrality of the system. The whole system was energy-minimized using steepest descent method up to maximum of 500 steps and production runs were performed. In cases where some lipids remained apart from the lipid bilayer, CG water particles were used to replace them and the whole system was again energy-minimized. These systems or the final states of self-assembly were subsequently prolonged under the same conditions as self-assembly simulations. All simulations were repeated 3–5 times.

The final configurations of four selected systems were used as inputs for the pulling experiments. The simulation box was extended in the *z* direction to capture the proposed trajectory of the pulling. Additional CG water particles were added to this extended space. The extended system was energy-minimized and short simulation for 50 ns was run. The CP was extracted from the membrane by applying a constraint force to the centre of mass (COM) of the protein in a direction coincident with *z* axis. Lipid molecules were restrained by position restraints during the pulling experiment (k_pr_ = 1000 kJ mol^−1^ nm^−2^). CP was pulled at a rate of 0.5 nm ns^−1^ and COM pulling was carried out until the COM of CP was 4 nm apart from COM of the lipid bilayer. Snapshots along the pulling trajectory were extracted at COM spacing of 0.1 nm to generate starting configurations for umbrella sampling windows. For umbrella sampling calculation, we used approx. 40 windows from the pulling experiment described above. All generated configurations (windows) were equilibrated for 50 ns before PMF calculation. Afterwards, for each window a 100 ns long simulation was performed with the biasing potential applied to restrain COM of CP in a required distance from COM of the lipid bilayer. PMF curves were obtained using the WHAM algorithm [51].

It is important to note that times reported in this study are computational times. It was shown that effective times for CG simulations are longer; for proteins and lipids in MARTINI force field, the speed up factor is about four-fold [52], i.e. 500 ns simulation time would correspond to 2 µs real time.

### Preparation of recombinant protein, purification, lipid-binding assays

The C-terminus of AtCP α subunit (AtCPα-Cterm, aa 270–308) was amplified by PCR using Phusion DNA polymerase (Finnzymes) and cloned into the pGEX-KG vector. The resulting plasmid (GST–AtCPα-Cterm) was transformed into *Escherichia coli* strain BL21 and cells were grown overnight at 37°C. After sub-culturing into fresh medium, cells were grown at 37°C to an OD_600_ of approximately 1.5, then induced for 4 h with 0.4 mM isopropyl thio-β-D-galactoside. Recombinant proteins were purified on glutathione-Sepharose (GE Healthcare) according to the manufacturer's instructions. Protein-lipid overlay assays with membrane lipid strips (Echelon) were performed according to manufacturer's instructions with protein concentration 0.5 µg/ml. To detect lipid binding in vesicles, we used the procedure described by [18] with slight differences; binding buffer comprised 125 mM KCl, 25 mM Tris, pH 7.8, 1 mM dithiothreitol and 0.5 mM EDTA. To reveal lipid binding, we incubated 400 nmol of lipids with 1 µg of GST-tagged protein.

## Supporting Information

Figure S1 **Docking of diacetyl-PA to AtCP.** The molecular docking was carried out using Autodock4 program [57]. To perform the docking of diacetyl-PA to AtCP, we utilized a procedure similar to that described by Kim et al. [11]. This figure was prepared using PyMol (http://www.pymol.org).
(TIF)

Figure S2 **Comparison of interaction of AtCP and GgCP with distinct membranes at 500 ns.** **A** The final state of the system containing AtCP – 50% POPA (charge −1)/POPC, **B** AtCP – 1% POPIP_2_/POPC, **C** GgCP – 1% POPIP_2_/POPC and **D** GgCP – POPC. Water molecules and Na^+^ ions are not shown for a sake of clarity. Headgroups and glycerol backbone atoms of POPIP_2_ and POPA are highlighted in van der Waals representation. AtCP is colored green and GgCP is in blue, only backbone atoms are shown in licorice representation. This figure was prepared using VMD [54].
(TIF)

Figure S3 **Density profile of the system containing AtCP – 20% POPA/POPC (A) and AtCP – 5% POPIP_2_/POPC (B).** The grey line represents water, green line AtCP, the blue line lipid tail atoms of POPA, POPIP_2_ and POPC. The two red lines represent headgroup and glycerol atoms of POPA, POPIP_2_ and POPC. The green line in enclosed graphs represents the α tentacle and the blue line stands for lipid tails.
(TIF)

Figure S4 **Sequence comparison of C-terminal parts of CPβ from different species.** The mafft algorith [40] was used to construct multiple alignments and the final figure was produced using the Jalview alignment editor [56]. Abbreviations used: At – *Arabidopsis thaliana*, Gg – *Gallus gallus*, Hs – *Homo sapiens*, Mb – *Monosiga brevicollis*, Os – *Oryza sativa*, Pp – *Physcomitrella patens*, Sc – *Saccharomyces cerevisiae*, Sm – *Selaginella moellendorffii*, Sp – *Schizosaccharomyces pombe*.
(TIF)
