# Supplementary figures and images for: Structural Insights into the Inhibition of Actin-Capping Protein by Interactions with Phosphatidic Acid and Phosphatidylinositol (4,5)-Bisphosphate
Source: PLoS Comput Biol. 2012 Nov 1;8(11):e1002765. doi: 10.1371/journal.pcbi.1002765 (PMC3486809; doi:10.1371/journal.pcbi.1002765)

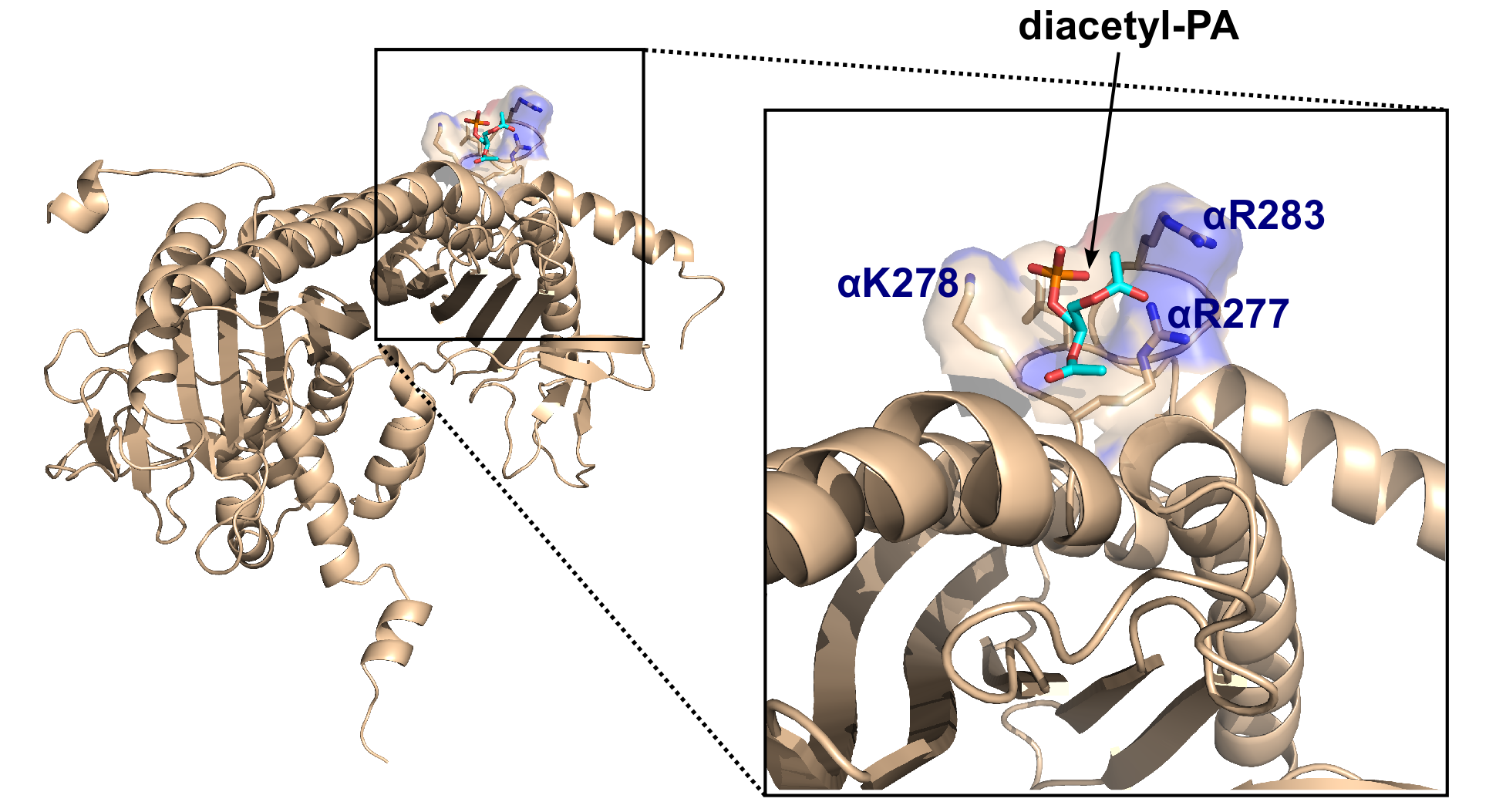

Supplement: Figure S1 — Docking of diacetyl-PA to AtCP. The molecular docking was carried out using Autodock4 program [57]. To perform the docking of diacetyl-PA to AtCP, we utilized a procedure similar to that described by Kim et al. [11]. This figure was prepared using PyMol (http://www.pymol.org). (TIF) [file pcbi.1002765.s001.tif]

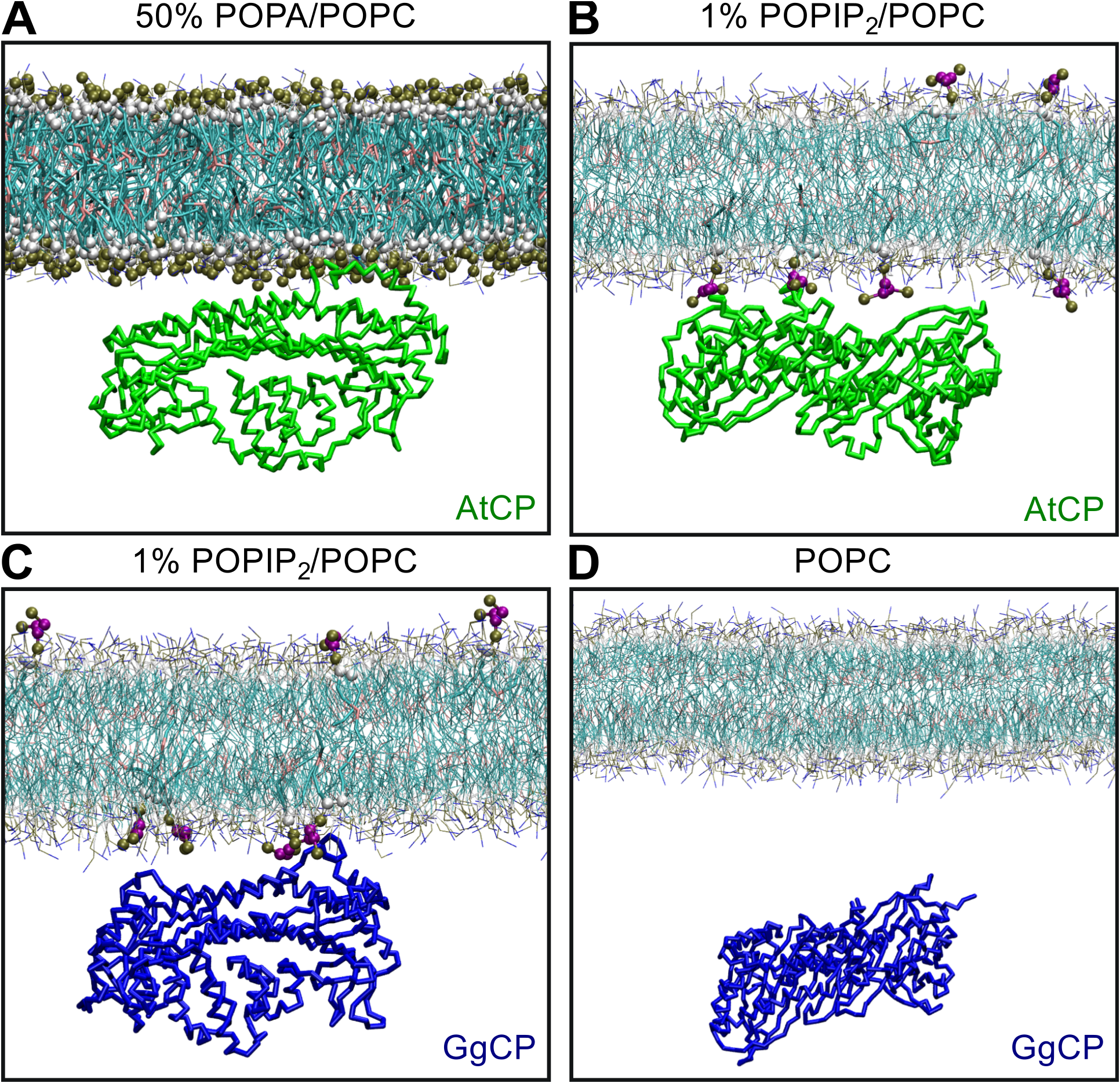

Supplement: Figure S2 — Comparison of interaction of AtCP and GgCP with distinct membranes at 500 ns. A The final state of the system containing AtCP – 50% POPA (charge −1)/POPC, B AtCP – 1% POPIP2/POPC, C GgCP – 1% POPIP2/POPC and D GgCP – POPC. Water molecules and Na+ ions are not shown for a sake of clarity. Headgroups and glycerol backbone atoms of POPIP2 and POPA are highlighted in van der Waals representation. AtCP is colored green and GgCP is in blue, only backbone atoms are shown in licorice representation. This figure was prepared using VMD [54]. (TIF) [file pcbi.1002765.s002.tif]

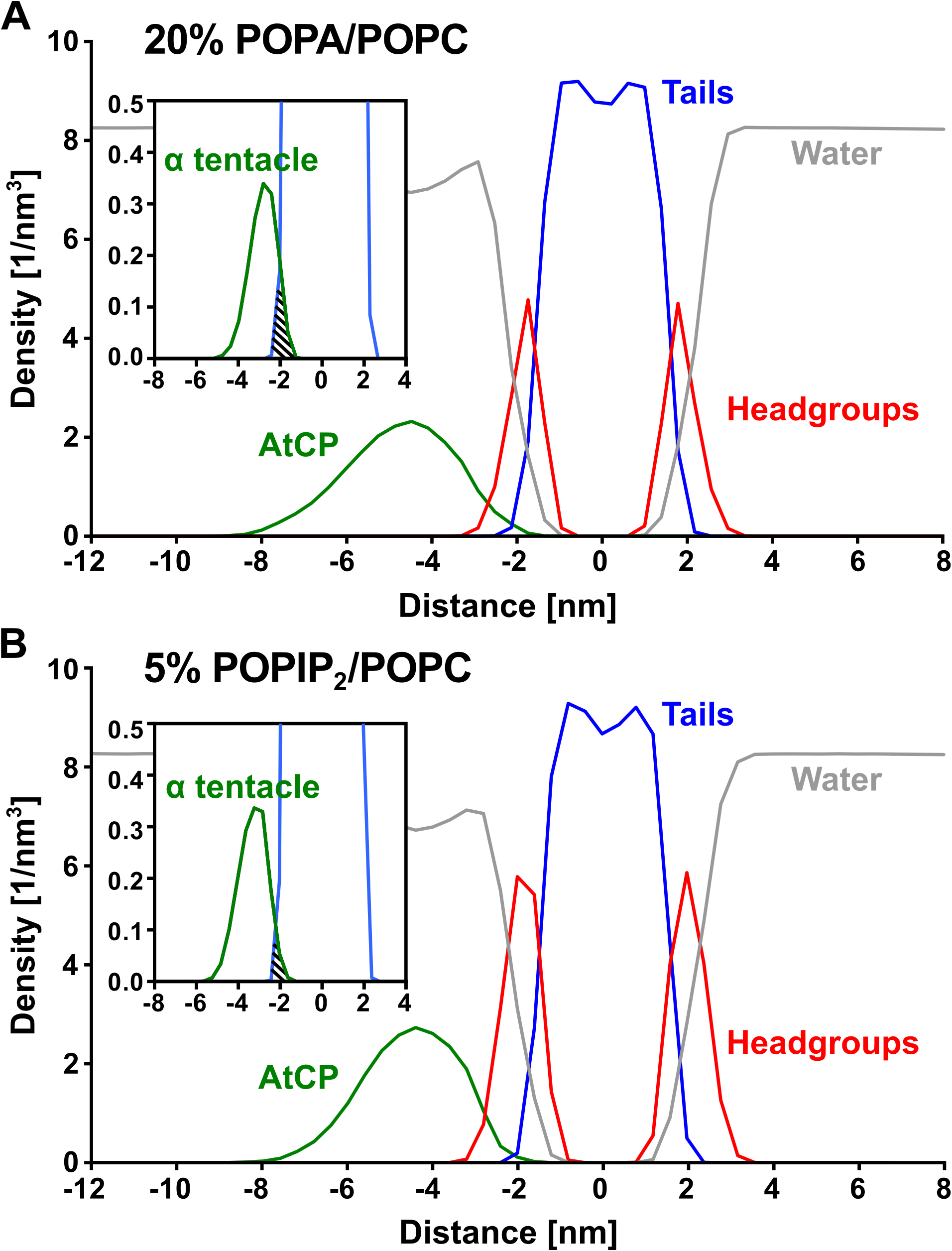

Supplement: Figure S3 — Density profile of the system containing AtCP – 20% POPA/POPC (A) and AtCP – 5% POPIP2/POPC (B). The grey line represents water, green line AtCP, the blue line lipid tail atoms of POPA, POPIP2 and POPC. The two red lines represent headgroup and glycerol atoms of POPA, POPIP2 and POPC. The green line in enclosed graphs represents the α tentacle and the blue line stands for lipid tails. (TIF) [file pcbi.1002765.s003.tif]

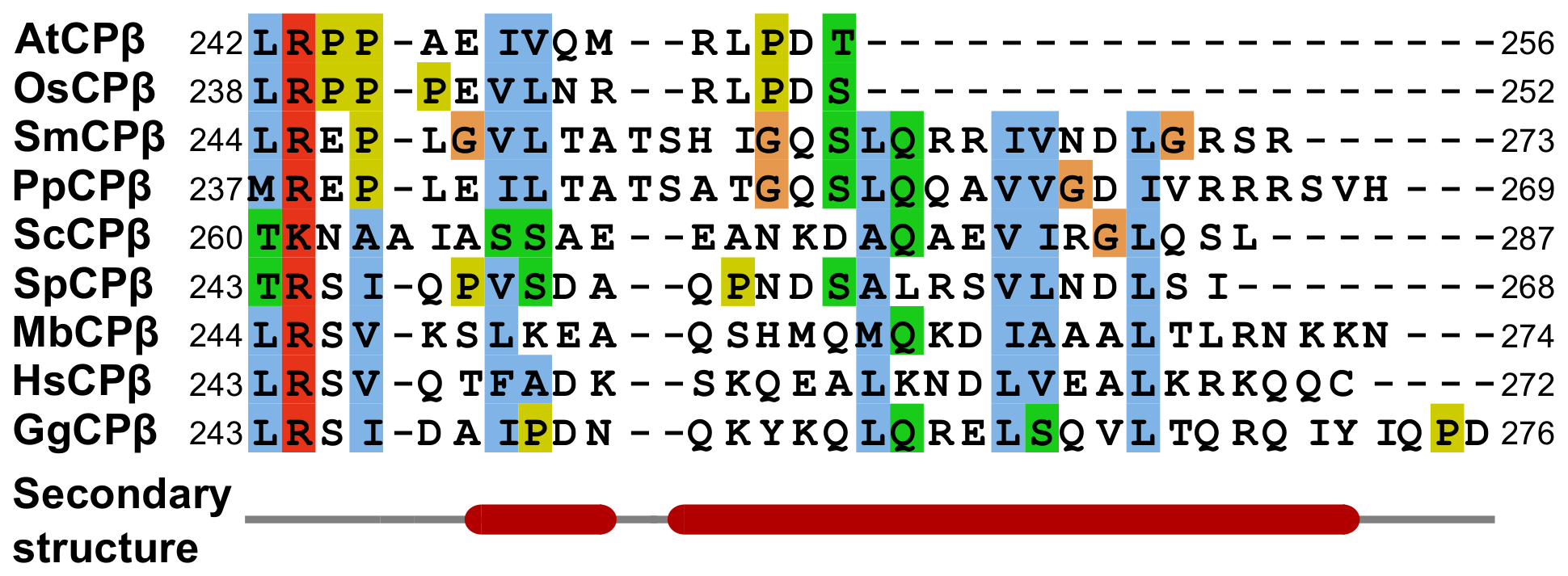

Supplement: Figure S4 — Sequence comparison of C-terminal parts of CPβ from different species. The mafft algorith [40] was used to construct multiple alignments and the final figure was produced using the Jalview alignment editor [56]. Abbreviations used: At – Arabidopsis thaliana, Gg – Gallus gallus, Hs – Homo sapiens, Mb – Monosiga brevicollis, Os – Oryza sativa, Pp – Physcomitrella patens, Sc – Saccharomyces cerevisiae, Sm – Selaginella moellendorffii, Sp – Schizosaccharomyces pombe. (TIF) [file pcbi.1002765.s004.tif]
